# The relationship between hearing loss and frailty in older adults at risk of cognitive decline: a cross-sectional study

**DOI:** 10.3389/fragi.2025.1524186

**Published:** 2025-03-24

**Authors:** Rong Tian, Osvaldo P. Almeida, Andrew H. Ford, Leon Flicker, Nicola T. Lautenschlager, Suzanne Robinson, Marshall Makate, Simone Pettigrew, Sin Huey Lee, Ina Dorsheimer, Jessica M. Yiannos, Libby Crawford, Dona M. P. Jayakody

**Affiliations:** ^1^ Medical School, University of Western Australia, Perth, WA, Australia; ^2^ WA Centre for Health and Ageing, Medical School, University of Western Australia, Perth, WA, Australia; ^3^ Department of Psychiatry, Faculty of Medicine, Dentistry and Health Sciences, The University of Melbourne, Melbourne, VIC, Australia; ^4^ Older Adult Mental Health Program, Royal Melbourne Hospital Mental Health Service, Parkville, VIC, Australia; ^5^ Deakin Health Economics, Institute for Health Transformation, Deakin University, Melbourne, VIC, Australia; ^6^ enAble Institute, Curtin University, Perth, WA, Australia; ^7^ Department of Health Economics and Data Analytics, Curtin University, Perth, WA, Australia; ^8^ The George Institute for Global Health, University of New South Wales, Sydney, NSW, Australia; ^9^ Ear Science Institute Australia, Subiaco, WA, Australia; ^10^ Murdoch University, Perth, WA, Australia; ^11^ Ear Sciences Centre, Faculty of Health and Medical Sciences, University of Western Australia, Perth, WA, Australia

**Keywords:** hearing loss, frailty, hearing handicap, central auditory processing, aging

## Abstract

**Objectives:**

To investigate the association between hearing loss and frailty among a group of older community volunteers with mild cognitive impairment.

**Design:**

This study recruited 162 older community volunteers who have mild cognitive impairment and symmetric age-related hearing loss. Participants’ hearing ability (including peripheral hearing, hearing handicap and central auditory processing) and frailty status were assessed and analysed. An independent t-test was conducted to compare hearing performance between frail and non-frail groups.

**Results:**

There were statistically significant differences between frail and non-frail groups for speech frequency hearing threshold, overall central auditory processing score and hearing handicap score, but not for high frequency hearing threshold.

**Conclusion:**

Frail individuals exhibit poorer performance in peripheral and central hearing assessments, as well as in self-reported hearing handicap. Future randomised controlled trials are necessary to find out if the correction of hearing loss decreases the proportion of people affected by frailty in later life.

## 1 Introduction

Frailty is a common clinical syndrome among older adults, reportedly affecting 3.5%–27% community-dwelling older adults in the Asia-pacific region, and over 50% in the socioeconomically disadvantaged and indigenous communities (Dent et al., 2017). This high variability can be attributed to different definitions of frailty and variations in sampling from the population (Dent et al., 2017). Currently, there are three major conceptual models of frailty: 1) the physical phenotype, developed by Fried et al., which describes frailty as a series of syndromes involving slowing, weakness, low energy and low activity (Fried et al., 2001); 2) the deficits accumulation model, developed by Rockwood and Mitnitsk, which conceptualises frailty as a multidimensional syndrome resulting from the accumulation of deficits across various domains of health and functioning (Mitnitski et al., 2001); and 3) mixed physical and psychosocial frailty models, which have gained more interest in recent years and several definitions have been established. Numerous tools have been developed based on these three models to measure frailty (de Vries et al., 2011). Each tool has its advantages and disadvantages and was developed to best fulfil the aim of measurement in clinical practice or research studies. In this present study, we focused solely on physical frailty only. Two widely used assessment tools, the Fried frailty phenotype and the FRAIL scale, were used. The Fried Frailty Phenotype is based on the physical phenotype model, while the FRAIL Scale is a simple, highly validated screening tool that combines elements of both the physical phenotype model and the deficits accumulation model.

While consensus on the best definition of frailty is lacking, there is agreement on the value of screening for it (Dent et al., 2017; Rodriguez-Manas et al., 2013). Frail individuals are more vulnerable to the deleterious effects of stressors and have an increased risk of dependency and mortality (Dent et al., 2017). In addition to negatively impacting the quality of life of older adults, healthcare expenses for frail individuals are about four times higher than those for their non-frail counterparts, imposing a significant burden on healthcare systems (Dent et al., 2017).

Several factors have been associated with frailty, including age-related physiological degeneration, multimorbidity, inflammation, sarcopenia, polypharmacy, endocrine disorders, protein energy malnutrition, social isolation, and poverty (Dent et al., 2017). Effective management of frailty risk factors, for example, through exercise, protein-calorie and vitamin D supplementation, and reduction of polypharmacy, holds potential for preventing or treating frailty effectively (Morley et al., 2013). Another emerging factor linked to increased risk of frailty is hearing loss, which ranks as the third leading cause of disability in the world (World Health Organization, 2021). Hearing loss has been reported to be linked to many frailty-related factors, including falls (Kamil et al., 2016), poor physical (Tareque et al., 2019) and psychosocial health (Almeida et al., 2019), dementia (Ford et al., 2018), and impaired activities of daily living (Tareque et al., 2019). Given its association with various frailty-related factors, hearing loss is likely associated with frailty. Studies investigating the association between hearing loss and frailty have emerged in recent years, however the available evidence remains inconclusive. That is, whilst reported findings mostly support the relationship between frailty and hearing loss, inconsistent results have been reported (Tian et al., 2021; Tan et al., 2020). It would be valuable to add further evidence to support this association. If hearing loss is indeed a risk factor of frailty, addressing such impairment could potentially reduce the risk or severity of frailty.

In a previous systematic review, the pooled results indicated that hearing loss was associated with an 87% increase in the risk of frailty among cross-sectional studies and 56% among longitudinal studies. However, the included studies varied significantly in study design, particularly in the measures used for hearing loss and frailty (Tian et al., 2021). The information regarding hearing loss in the available literature on this topic is not comprehensive. Many studies published to date have relied on self-reported hearing loss as the relevant exposure for frailty, and have predominantly focused on the role of peripheral hearing loss, which along with central auditory processing (CAP) constitute the two main components of the auditory system (Tian et al., 2021). Peripheral hearing ability refers to sound detection, whereas CAP is involved in speech comprehension, especially in the presence of background noise or competitive speech (Sardone et al., 2021). Given that both peripheral and central auditory abilities decline with age, the investigation of hearing loss and frailty should include the assessment of both components (Nuesse et al., 2021). Furthermore, hearing handicap, which describes the impact of hearing loss on an individual’s daily life, should also be considered, as it is influenced by various physical, mental, and social factors (Nuesse et al., 2021).

The existing evidence regarding the relationship between hearing loss and frailty is neither conclusive nor comprehensive. Therefore, the aim of the present study is to investigate the association between hearing loss and frailty in older adults using audiological measures of peripheral hearing and CAP, as well as subjective self-reported hearing handicap. By examining multiple components of hearing loss and their association with frailty, we aim to enhance our understanding of their relationship.

## 2 Materials and methods

### 2.1 Study design and setting

This study employed a cross-sectional design and involved a sample of older community volunteers residing in Perth, Western Australia. Participants were recruited for the HearCog trial, and this study reports data derived from the baseline assessment. Further details about the study design and procedures have been published (Jayakody et al., 2020a). Hearing assessments were performed by qualified audiologists. Other assessments were conducted by trained research staff at the research centre. Questionnaires were predominantly self-administered and completed by participants during research appointments with guidance from study staff, and assistance was provided when necessary. Frailty status was calculated after all data collection were completed, therefore, audiologists and researchers were blinded to participants’ frailty status at the time of assessments. The Human Research Ethics Committee of the University of Western Australia approved the research protocol and related activities, and written consent was obtained from all participants.

### 2.2 Participants

The study included older adults aged 70 years and older who were recruited through Ear Science Institute of Australia and Lions Hearing Clinics, as well as retirement villages, radio, newspaper, and social media advertisements. Participants were required to have symmetric age-related hearing loss, defined as better ear average hearing loss greater than 23.3 dB HL at 0.5, 1, and 2 kHz, or high-frequency average hearing loss of 40 dB HL or greater at 2, 3, and 4 kHz, in accordance with the Australian Hearing Services Program Guideline (Australian Government Department of Health and Aged Care, 2022), and have no prior exposure to the use of hearing aids. This study is a secondary analysis of the HearCog trial, which investigated the efficacy of hearing aids on cognitive function, therefore, participants originally recruited for the trial were required to have mild cognitive impairment (MCI) (scored greater than 18 and less than 26 on the Montreal Cognitive Assessment for the Hearing Impaired, HI-MoCA (Lin et al., 2017)).

### 2.3 Study measures

#### 2.3.1 Assessment of hearing

Peripheral hearing was assessed using pure-tone audiometry (PTA) with a clinical audiometer (MIDIMATE 602 Audiometer, GN Otometrics Ltd., Sydney) and supra-aural earphone. Qualified audiologists conducted bilateral air-conduction thresholds measurements at 0.25, 0.5, 1, 2, 4, 6, and 8 kHz in a soundproof booth at the Ear Science Institute Australia. In cases where baseline PTA data were unavailable (n = 15), data from the KUDUwave (KUDUwave™ 5,000 Plus, GeoAxon Global Ltd., South Africa) were used instead. KUDUwave is a portable diagnostic and screening audiometer that has been clinically validated. For the statistical analysis of the study, the average air conduction thresholds of the better ear across 0.5–4 kHz were considered speech-frequency hearing thresholds, while the average air conduction thresholds of the better ear at 4, 6, and 8 kHz were considered as high-frequency hearing thresholds.

Self-reported hearing handicap was assessed by the Hearing Handicap Inventory of the Elderly (HHIE) (Ventry and Weinstein, 1982). The HHIE is a 10 items questionnaire assessing emotional and social impact of one’s perceived hearing difficulties. Scores on this inventory range from 0 (no handicap) to 40 (maximum handicap).

The assessment of CAP included three tests. To simplify the analysis and reduce the risk of multiple comparisons, an overall CAP score was calculated using principal component analysis based on the results of these tests. The Dichotic Digits Test (DDT) evaluated the binaural integration (Musiek et al., 1991). Participants were presented with two digits in each ear simultaneously and asked to repeat all four digits to the best of their ability. The Synthetic Sentence Identification with Ipsilateral Competing Message (SSI-ICM) assessed participants’ ability to identify 10 short nonsense sentences against a background competing signal (Orchik and Burgess, 1977). Scores were based on the proportion of correct identification of sentences (0%–100%). The scores of the better ear at message-competition ratio of 0 dB were used for the analysis. Finally, the Quick Speech in Noise (Quick-SIN) measured participants’ ability to hear in a context of background noise (Killion et al., 2004). Participants were presented with two practice sentences and two sets of six test sentences for each ear. These sentences were presented with multi-talker babble noise, with signal-to-noise ratios of 25, 20, 15, 10, 5 and 0 dB. Each sentence contained five keywords, and participants scored one point for each correctly repeated word. The total score was subtracted from 25.5 to calculate the signal-to-noise ratio loss. A higher score indicates poorer speech understanding ability with background noise.

#### 2.3.2 Assessment of frailty

We used two validated measurement tools of frailty: the FRAIL scale and Fried frailty phenotype. Participants were classified as frail if they met the frailty criteria in either of these two measures; otherwise, they were classified as non-frail.

The FRAIL scale (Abellan van Kan et al., 2008; Morley et al., 2012a) assessed five relevant domains: fatigue, resistance, ambulation, illness, and loss of weight. Participants who scored positive in one or two domains of the FRAIL scale were considered pre-frail, and those score more than two were considered frail. Fatigue was determined by participants’ response to the question, “how much of the time during the past 4 weeks have you felt tired or worn out?” Possible answers ranged from “all or most of the time” (positive) to “a little or none of the time” (negative). To assess resistance, ambulation, and loss of weight, participants answered “yes” or “no” to questions about difficulty walking up to 10 steps without resting in the past 4 weeks, difficulty walking 200 m or one block without aids in the past 4 weeks, and weight loss of more than 5 kg or 5% of body weight in the past year, respectively. The presence of illness was rated as present if participants reported having five or more of the following conditions: hypertension, diabetes, cancer (other than minor skin cancer), chronic lung disease (chronic bronchitis or emphysema), heart attack, congestive heart failure, angina, asthma, arthritis, stroke, and kidney disease (Morley et al., 2012b).

The Fried frailty phenotype had five components: unintentional weight loss, weakness, exhaustion, slow walking speed, and low physical activity (Fried et al., 2004). Participants who exhibited one or two components were defined as pre-frail, and those with three or more were considered to be frail. Unintentional weight loss and exhaustion was assessed using the same questions for weight loss and fatigue as the FRAIL scale. Weakness was defined as grip strength less than 26 kg for men or less than 18 kg for women (Gu et al., 2019), which was measured using a Jamar Smart Hand Dynamometer. Walking speed was self-reported, with participants selecting one of the following options to describe their usual unaided walking speed: “fast or fairly brisk”, “normal speed for my age”, “slightly slow”, “very slow”, or “unable to walk independently”. The walking speed of participants was deemed impaired if they responded “slightly slow, very slow, or unable to walk independently”. Physical activity was assessed using two self-reported questions: “In a usual week, do you do any non-vigorous exercise for recreation or health and fitness?” and “In a usual week, do you do any non-vigorous exercise for recreation or health and fitness?” Participants who reported no exercise (non-vigorous or vigorous) in a usual week were classified as having low physically activity.

#### 2.3.3 Other measurements

Demographic and lifestyle information was collected using a questionnaire (summarised in Table 1). We calculated the age of participants (in years) by subtracting the date of birth from the date of the assessment and then dividing the result by 365.25. Participants were categorized according to whether they had completed a least 12 years of education. Smoking status was classified as never, former, or current. Risky drinking was defined by the consumption of four or more standard alcoholic drinks on any given day or 10 or more drinks in a usual week (Australian Institute of Health and Welfare, 2021). The Patient Health Questionnaire (PHQ-9) was administered to assess depressive symptoms, with a total score of 10 or greater indicating the presence of clinically significant symptoms of depression (Kroenke et al., 2001).

**TABLE 1:** Sociodemographic and clinical characteristics of non-frail and frail participants. Group differences were assessed using t-tests for continuous variables and Chi-squared (χ^2^) tests for categorical variables. The associated p-values are presented.

Characteristics		Non-frail N = 151 n (%) or mean ± SD	Frail N = 11 n (%) or mean ± SD	χ2/t	p-Value
Age (years)	70–75	64 (42.4)	2 (18.2)	3.30	0.347
	75–80	48 (31.8)	5 (45.5)		
	80–85	27 (17.9)	2 (18.2)		
	>85	12 (8.0)	2 (18.2)		
Gender	Female	54 (35.8)	7 (63.6)	3.39	0.065
	Male	97 (64.2)	4 (36.4)		
Minimum of 12 years of education	Yes	51 (33.8)	1 (9.1)	2.87	0.090
	No	100 (66.2)	10 (90.9)		
Marital status	Never married	2 (1.3)	1 (9.1)	19.28	<0.000
	Married/Defacto	117 (77.5)	2 (18.2)		
	Separated/Divorced/Widowed	32 (21.2)	8 (72.7)		
Smoking Status	Never	81 (53.6)	6 (54.6)	1.03	0.596
	Former	65 (43.1)	4 (36.4)		
	Current	5 (3.3)	1 (9.1)		
Risky alcohol use	No	134 (88.7)	11 (100.0)	1.38	0.239
	Yes	17 (11.3)	0 (0.0)		
Depression	No	146 (96.7)	9 (81.8)	5.48	0.019
	Yes	5 (3.3)	2 (18.2)		
MoCA		22.5 ± 2.03	21.1 ± 2.06	2.29	0.023

^a^ Abbreviations: SD: standard deviation; MoCA: the Montreal Cognitive Assessment.

### 2.4 Statistical analyses

An initial exploratory analysis was conducted to compare hearing performance between frailty groups. The methods and results of this analysis were described in the Supplementary Material.

The data analysis was conducted using the statistical software Stata 16.0 (StataCorp LLC, 2019). Descriptive statistics were used to summarise continuous data using mean, standard deviation (SD), and range, while categorical variables were presented as counts and percentages (%). To calculate the overall CAP score for each participant, a factor analysis with principal component extraction was performed using the test scores of DDT, SSI-ICM, and Quick-SIN. An eigenvalue threshold greater than one was used in the factor analysis, and the resulting loading scores were used as an overall summary measure of CAP. Pearson chi-squared tests were used to investigate relationships between sociodemographic and lifestyle characteristics of non-frail and frail participants. An independent t-test was conducted to compare hearing measures between groups.

We completed a series of *post hoc* analyses using independent t-tests. We examined the association between each of three CAP tests score and frailty. In addition, to assess whether the association between hearing loss and frailty is influenced by the severity of frailty, we introduced a new pre-frail/frail group by combining participants who were pre-frail according to either the FRAIL scale or the Fried frailty phenotype with those who were frail. This new classification is different from that in the primary analysis, where both pre-frail and normal participants were categorised as non-frail. Hearing measures of this pre-frail/frail group were compared with normal group using t-tests. Alpha was set at 5% and all probability tests reported are two-tailed.

## 3 Results

We recruited 162 participants, with a mean age of 77.2 years (SD 5.2, range 69.8–92.7). Sociodemographic and clinical characteristics according to frailty status are presented in Table 1. In total, 11 (6.8%) participants were frail according to either the FRAIL scale or Fried frailty phenotype. Fried frailty phenotype identified more frail participants (n = 11, 6.8%) than the FRAIL scale (n = 3, 1.9%).

Individuals in the frail groups exhibited poorer performance in all three measures of hearing compared to the non-frail group (Table 2). Independent t-tests showed statistically significant difference between frail and non-frail groups for speech frequency hearing threshold [t (160) = −3.40 p = 0.001], overall CAP score [t (160) = 2.64 p = 0.009], and HHIE score [t (160) = −2.16 p = 0.032], but not for high frequency hearing threshold (Table 2).

**TABLE 2:** Relationship between hearing measures and frailty status. Independent t-test results and p-values are presented.

	Non-frail N = 151		Frail N = 11		t-test	p-Values
	Mean	SD	Mean	SD		
Speech frequency hearing threshold	36.17	7.10	43.98	10.40	−3.40	0.001
High frequency hearing threshold	60.83	12.19	68.03	13.78	−1.87	0.063
Overall CAP score	0.05	0.85	−0.65	0.87	2.64	0.009
HHIE score	18.03	8.06	23.45	7.90	−2.16	0.032
DDT score	84.42	12.50	75.09	17.38	2.32	0.021
Quick-SIN score	10.65	5.00	13.45	4.79	−1.80	0.074
SSI-ICM score	73.91	26.28	48.18	25.62	3.14	0.002

^a^ Abbreviations: SD: standard deviation; CAP: central auditory processing; HHIE: hearing handicap inventory of the elderly; DDT: dichotic digits test; Quick-SIN: quick speech in noise; SSI-ICM: synthetic sentence identification with ipsilateral competing message.

### 3.1 Post-hoc analyses

Further *post hoc* analyses investigating the association between each individual CAP measures and frailty status showed a statistically significant difference between frailty groups for the SSI-ICM [t (160) = 3.14, P = 0.002] and DDT [t (160) = 2.32, P = 0.021], but not QuickSIN [t (160) = −1.80, P = 0.074] (Table 2).

When combining the pre-frail and frail participants, 76 (46.9%) were pre-frail/frail. We found no significant difference in any hearing measures between the pre-frail/frail and normal groups (Table 3).

**TABLE 3:** Hearing measures of normal and pre-frail/frail participants. Independent t-test results and p-values are presented.

	Normal N = 86		Pre-frail/frail N = 76		t-test	p-Values
	Mean	SD	Mean	SD		
Speech frequency hearing threshold	36.18	0.82	37.29	0.86	−0.93	0.355
High frequency hearing threshold	60.71	1.32	62.02	1.44	−0.67	0.504
Overall CAP score	0.07	0.09	−0.08	0.11	1.08	0.281
HHIE score	18.21	0.87	18.61	0.95	−0.31	0.758

^a^ Abbreviations: SD: standard deviation; CAP: central auditory processing; HHIE: hearing handicap inventory of the elderly.

## 4 Discussion

Our study examined the relationship between frailty and relevant measures of hearing in a sample of older adults with mild cognitive impairment. We found that frail participants had worse hearing compared with their non-frail peers. Specifically, a statistically significant difference was observed between groups on speech-frequency hearing thresholds, overall CAP, and self-reported hearing handicap.

We acknowledge that our sample size was modest and may have been subject to bias. The study included older adults from Perth, Western Australia, who volunteered to join the study. It is unclear how well they represent the population of older adults living in the community, particularly because they all had mild cognitive impairment and some level of hearing loss. Hence, the generalisability of our findings is uncertain. Mild cognitive impairment is the term given to patients with cognitive impairment that is detectable by clinical criteria but does not produce impairment in daily functioning (Petersen, 2004). The mean MoCA score of participants was 22.3 (range 18–25). Participants scored within this range were classified as having MCI but expected to maintain normal daily functioning ability. While there is a risk that the accuracy of self-reported questionnaires could be affected by the MCI, we do not anticipate it significantly impact the quality of results. Similarly, the frail group (mean = 21.1, SD = 2.06) scored slightly lower on the MoCA compared to the non-frail group (mean = 22.5, SD = 2.03), although the imbalance between the groups could potentially introduce bias into our results, we do not expect significant impact as the difference is relatively small, and MCI should not substantially affect participants' performance. In addition, our investigation had a cross-sectional design, making it difficult to determine with confidence the direction of the association between hearing loss and frailty.

We utilised validated tools to measure both frailty and hearing loss. Our measures of frailty are well-validated and widely used. To increase the sensitivity of our measurements, we classified frailty as identified by either the FRAIL scale or Fried frailty phenotype. The 6.8% prevalence of frailty reported in this study is lower than that in other studies, which reported a pooled prevalence of 9.9% for physical frailty among community-dwelling adults aged 65 and older (Collard et al., 2012). Several other studies using the FRAIL scale (Tian et al., 2022) or Fried frailty phenotype (Castellana et al., 2021; Herr et al., 2018) also reported an association between hearing loss and frailty, giving some face-validity to our findings, although contrasting findings (Gu et al., 2019) also exist.

A strength of our study was the detailed assessment of hearing loss, which included a comprehensive evaluation of both peripheral and central hearing, and the subjective impact of hearing loss. We used PTA to examine peripheral hearing. PTA is considered a “gold” standard for hearing thresholds assessment, as it can provide diagnosis and detailed information regarding the degree and spectrum of hearing loss. We analysed both speech-frequency and high-frequency hearing thresholds, although the results for speech-frequency hearing thresholds had a narrow distribution, which limited the inferences that could be drawn from the data. Furthermore, most participants had mild hearing loss, and such a level of impairment may not have been sufficiently severe to demonstrate unequivocally its association with frailty, an issue that has also been discussed by others (Kamil et al., 2016; Castellana et al., 2021). Of note, the association between CAP and physical frailty has rarely been studied, and the use of principal component analysis to generate an overall CAP score contributed to circumvent the issue of multiple comparisons. Additionally, both frailty and CAP are associated with cognitive function (Panza et al., 2015), and the presence of MCI in our sample may have attenuated the observed association between CAP and frailty. Cognitive impairment is associated with frailty and incorporated in certain frailty measures. As all participants had MCI, there is a possibility that they were already predisposed to frailty. If MCI serves as one of the pathways linking CAP and frailty, the comparison between groups may fail to capture the effect of CAP through this pathway, but rather the effect of other pathways. Nonetheless, we found that frail participants exhibited poorer overall CAP performance than their non-frail peers. Another strength of our study was the inclusion of a measure of self-reported hearing handicap, particularly because people with the same severity of hearing loss may experience different degrees of hearing disability (Chen, 1994). Finally, we acknowledge that residual error and confounding by unmeasured factors could potentially account for some of the observed associations, and the modest overall sample size and the imbalance between frail and non-frail groups may limit the statistical power of our analyses.

Our *post hoc* analyses revealed that there was no significant difference in any hearing measures between pre-frail/frail group and normal group, whereas we found frail group exhibited poorer performance in speech frequency hearing thresholds, HHIE scores, and CAP scores compared to the rest. This indicates that the association between hearing loss and frailty may be more pronounced among individuals with higher severity of frailty.

We analysed three aspects of hearing. Firstly, for peripheral hearing, we assessed speech-frequency and high-frequency hearing thresholds, and only found a statistically significant difference in speech-frequency hearing threshold between the frail and non-frail/groups. Similarly, Yevenes-Briones et al. (2021) only found an association with speech-frequency but not with high-frequency hearing loss. Liu et al. (2022) investigated both speech and high frequency hearing loss and reported that both were associated with frailty, while Sardone et al. (2021) reported no significant association between peripheral hearing loss (both low-middle and high frequency hearing thresholds) and frailty, although they did find a higher prevalence of peripheral hearing loss in the frail than the non-frail group. Hura and colleagues also reported a linear association between PTA results (both low and high frequency hearing thresholds) and the Frailty Index (Hura et al., 2022). Taken together, our results and those of others indicate that speech-frequency hearing thresholds are the hearing measures most consistently associated with frailty. While both speech and high frequency hearing ability are important, speech frequencies are critical for audibility, with high frequencies contributing towards speech clarity. Extended high frequency hearing loss has also gained interest in recent years, and its association with frailty may be an area for future research. It is believed to be a very early sign of age-related hearing loss and may play important role in speech perception and localisation (Hunter et al., 2020). Extended high-frequency hearing thresholds were not assessed in this study, as they are not part of routine clinical testing and established norms are lacking. Secondly, we examined the association between self-reported hearing handicap and frailty, an area that has received limited attention in previous studies. Two studies have reported an association between hearing handicap and frailty (Nuesse et al., 2021; Campos et al., 2022), with our results suggesting a non-compelling association. Thirdly, in the present study, we found a difference in the overall CAP score between frail and non-frail groups. When examining the three CAP tests separately, we found significant differences in SSI-ICM scores (p = 0.002). This discrepancy might be attributed to the SSI-ICM test’s higher sensitivity in assessing CAP in individuals with MCI (Jayakody et al., 2020b). In line with this, Sardone and colleagues reported no association between the SSI-ICM test results and physical frailty, but an association was found when a frailty model including both physical and cognitive components was utilised (Sardone et al., 2021). A difference was also found in DDT, but not in QuickSIN scores.

Several proposed pathways have been suggested to link hearing loss and frailty: shared underlying pathological process, such as inflammation markers and vascular factors (Kamil et al., 2016; Sardone et al., 2021; Castellana et al., 2021; Yevenes-Briones et al., 2021; Liu et al., 2022; Hura et al., 2022), communication limitations leading to social limitations and restrictions in daily living (Kamil et al., 2014; Liljas et al., 2017), and concurrent cognitive decline (Panza et al., 2015). Current evidence is insufficient to determine the mechanisms linking hearing loss and frailty, and there is no compelling evidence showing that the remediation of hearing loss decreases frailty.

We believe future research on the relationship between hearing loss and frailty would be valuable. Hearing loss is highly prevalent among older adults, affecting over 65% of adults aged 60 years and above (World Health Organization, 2021). If hearing loss is an early marker of frailty, regular screening for hearing loss should be established in geriatric settings to detect early signs of frailty. Furthermore, if hearing loss is a modifiable risk factor of frailty and future evidence supports that hearing remediation can manage frailty, then hearing loss management should be recommended to individuals at risk of or with frailty. Hearing aids are the most common treatment for hearing loss. Given the high prevalence of hearing loss and the cost-effectiveness and safety of treatment with hearing aids, the appropriate management of hearing loss could represent a valuable intervention to decrease the prevalence of frailty or alleviate its symptoms.

In summary, our study revealed that frail individuals exhibited poorer performance in assessments evaluating both peripheral and central hearing, as well as in self-reported hearing handicap. We identified statistically significant differences between frail and non-frail participants in speech-frequency hearing thresholds, overall CAP scores, and hearing handicap scores. However, the modest sample size may restrict the statistical power and limit the robustness of our findings. Future studies with larger sample sizes, or conducted in populations with higher frailty prevalence would be beneficial. Additionally, randomised controlled trials investigating whether correcting hearing loss reduces the proportion of people affected by frailty in later life would also be of interest.

## Data Availability

The raw data supporting the conclusions of this article will be made available by the authors, without undue reservation.

## Abbreviations

CAP, central auditory processing; MCI, mild cognitive impairment; PTA, pure-tone audiometry; HHIE, the Hearing Handicap Inventory of the Elderly; SSI-ICM, the Synthetic Sentence Identification with Ipsilateral Competing Message; Quick-SIN, the Quick Speech in Noise; DDT, the Dichotic Digits Test; MoCA, the Montreal Cognitive Assessment.
