# Supplementary material for: The relationship between hearing loss and frailty in older adults at risk of cognitive decline: a cross-sectional study
Source: Front Aging. 2025 Mar 24;6:1524186. doi: 10.3389/fragi.2025.1524186 (PMC11973386; doi:10.3389/fragi.2025.1524186)
Supplement: Supplementary file 1 [file DataSheet1.PDF]

Supplementary material 1

Methods:

In this initial exploratory analysis, participants were grouped as non-frail, pre-frail, and frail according to the FRAIL scale and Fried frailty phenotype. One way analysis of variance (ANOVA) was performed to test the association between hearing loss and overall frailty, followed by Scheffe correction for multiple-comparisons.

Results:

In this initial exploratory analysis, we found that participants in the less impaired groups (non-frail or pre-frail) performed better than their frail counterparts on most hearing measures. There were no obvious differences in any of the study measures between the non-frail and pre-frail groups (eFigure 1). One-way ANOVA showed a statistically significant difference between the group for speech-frequency hearing thresholds [$F(2,159)=3.67$, $P=0.028$], HHIE [$F(2,159)=3.33$, $P=0.039$], and overall CAP [$F(2,159)=3.84$, $P=0.024$], but not for high-frequency hearing thresholds [$F(2,159)=0.88$, $P=0.419$]. Post-hoc analysis using Scheffe correction for multiple-comparisons confirmed differences between frail and non-frail participants in speech-frequency hearing thresholds and overall CAP scores. The frail and pre-frail groups had different HHIE and overall CAP scores. See eTable 1 for details.

eFigure 1 Hearing measures according to frailty status

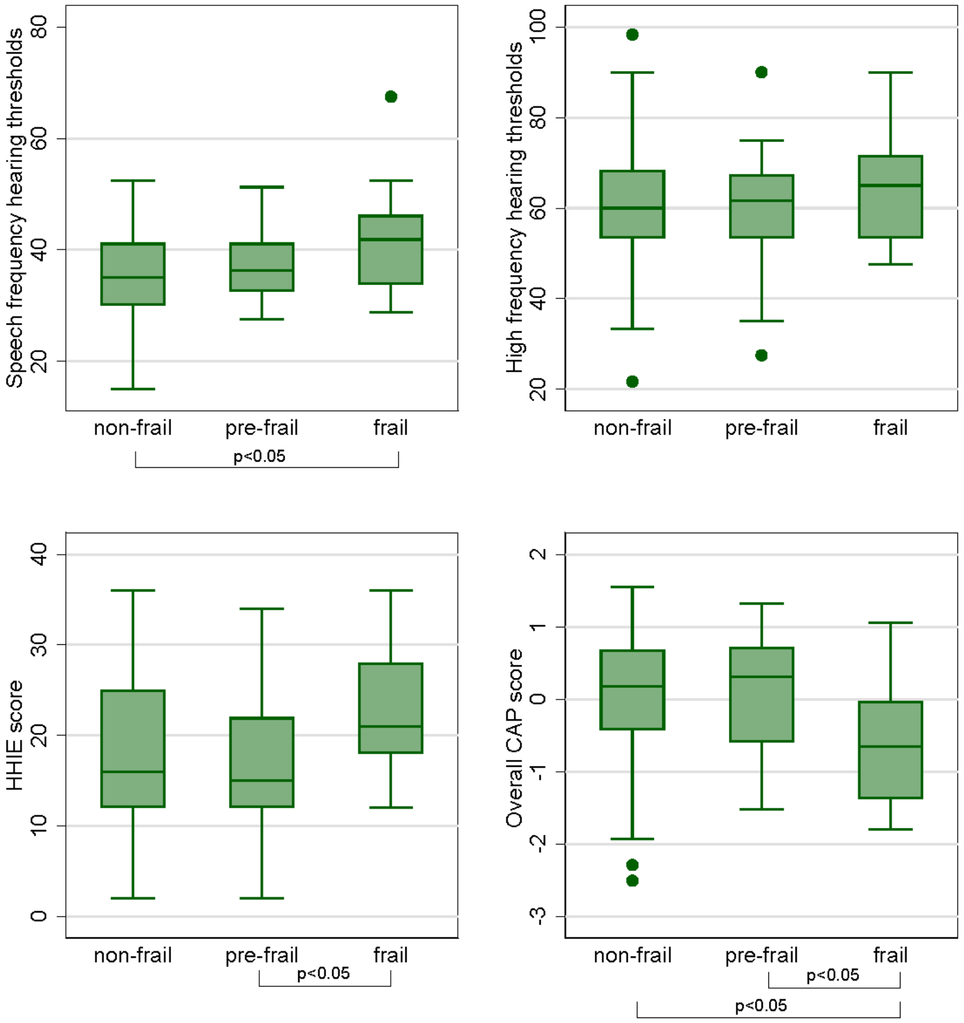

HHIE: the Hearing Handicap Inventory of the Elderly; CAP: central auditory processing

eTable 1 Results of One-Way Analyses of Variance and Scheffe correction for multiple-comparisons

	**One-Way Analyses of Variance**		**Scheffe correction for multiple-comparisons**		
	F(2, 159)	p-value	Non-frail VS Pre-frail MD (p-value)	Non-frail VS Frail MD (p-value)	Pre-frail VS Frail MD (p-value)
**Speech frequency hearing threshold**	3.67	0.028	0.76 (0.874)	5.40 (0.028)	4.64 (0.126)
**High frequency hearing threshold**	0.88	0.419	-1.17 (0.890)	3.75 (0.529)	4.92 (0.426)
**HHIE score**	3.33	0.039	-2.06 (0.426)	4.21 (0.148)	6.27 (0.039)
**Overall CAP score**	3.84	0.024	0.53 (0.950)	-0.60 (0.032)	-0.66 (0.041)

Note: CAP: MD: mean difference; central auditory processing; HHIE: Hearing Handicap Inventory of the Elderly.
